# Investigating the impact of collateral circulation pathways on hemodynamics in iliac vein compression syndrome

**DOI:** 10.3389/fbioe.2026.1812311

**Published:** 2026-06-10

**Authors:** Ke Hu, Peng Qiu, Xiaoya Wang, Jiaqiu Wang, Shuang Zhang, Xiaoxiao Yang, Yuanli Chen, Chenshu Li, Xinwu Lu, Kaichuang Ye, Yanqing Zhan, Lingling Wei

**Affiliations:** 1 Key Laboratory of Metabolism and Regulation for Major Diseases of Anhui Higher Education Institutes, Anhui Provincial International Science and Technology Cooperation Base for Major Metabolic Diseases and Nutritional Interventions, School of Food and Biological Engineering, Hefei University of Technology, Hefei, China; 2 Department of Vascular Surgery, Shanghai Ninth People’s Hospital Affiliated to Shanghai Jiao Tong University School of Medicine, Shanghai, China; 3 School of Engineering, London South Bank University, London, United Kingdom; 4 Department of Vascular Surgery, The Affiliated Chuzhou Hospital of Anhui Medical University, Chuzhou, China; 5 Department of General Surgery, The First Affiliated Hospital of Anhui Medical University, Hefei, China; 6 Anhui Public Health Clinical Center, Hefei, China

**Keywords:** collateral circulation, computational fluid dynamics, iliac vein compression syndrome, venous hemodynamics, wall shear stress

## Abstract

This study aims to investigate the hemodynamic effects of collateral circulation in Iliac Vein Compression Syndrome (IVCS) using computational fluid dynamics (CFD). Two patient-specific three-dimensional models were reconstructed from Computed Tomography Angiography (CTA) data: one with a well-developed collateral circulation system (collateral model) and the other without significant collateral development (non-collateral model). A porous medium model was applied to simulate venous spur-like obstruction within the compressed iliac vein segment. The Shear Stress Transport (SST) k-ω model was employed to simulate blood flow under physiological boundary conditions, incorporating the influence of gravity. Key hemodynamic parameters including blood reflux, pressure distribution, flow velocity, and wall shear stress (WSS), were systematically analyzed and compared between the two models. The results demonstrate that the presence of collateral circulation significantly altered and stabilized the hemodynamic environment. In the collateral model, minimal blood reflux was observed due to flow redistribution, resulting in a notably lower pressure gradient across the stenotic region (peak pressure difference: 619 Pa) and reduced WSS (peak WSS: 10 Pa). In contrast, the non-collateral model exhibited extensive reflux and large-scale vortex formation, an extreme pressure gradient (peak pressure difference: 20,190 Pa, approximately 32.6 times higher than that of the collateral model (619 Pa), and significantly elevated WSS (peak WSS: 56 Pa). Furthermore, the total venous outflow to the inferior vena cava in the collateral model was 39.38% higher than that in the non-collateral model, indicating enhanced venous return. These findings suggest that collateral circulation may play important compensatory hemodynamic role in IVCS by redistributing venous flow and reducing pressure burden, wall shear stress, and the extent of disturbed-flow structures in the compressed region. The present results should be interpreted as mechanistic and hypothesis-generating rather than as direct evidence of thrombotic risk or clinical treatment effect. Assessment of collateral pathways may nevertheless provide useful mechanistic insight into venous return in patient-specific IVCS anatomy.

## 1 Introduction

Iliac Vein Compression Syndrome (IVCS) is a pathological condition characterized by compromised venous return from the lower extremities and pelvis, which may arise from either external compression of the iliac vein or intrinsic structural abnormalities of the vessel (Kearon, 2003; Kalu et al., 2013; Meissner, 2007). This syndrome commonly occurs when the right common iliac artery crosses anteriorly over the left common iliac vein, resulting in pulsatile compression that is further exacerbated by the posterior lumbar spine. This anatomical configuration leads to narrowing of the venous lumen, restricted blood flow, and elevated venous pressure (Poyyamoli et al., 2021). In the context of the present study, the primary interest is not the broad clinical spectrum of IVCS, but rather the hemodynamic consequences of venous obstruction and collateral compensation from a computational fluid-dynamics perspective.

Collateral circulation is often observed in IVCS and may serve as a compensatory outflow pathway when the compressed iliac vein cannot adequately drain venous blood (Wang et al., 2014). Previous studies have suggested that collateral pathways may partially reduce venous pressure, improve local hemodynamics, and alleviate symptoms associated with venous outflow obstruction (Zürcher et al., 2022; Assi et al., 2023). However, the presence of collateral circulation alone does not fully explain the hemodynamic severity of IVCS, because patients with apparently similar anatomical compression may still exhibit markedly different flow disturbances.

Blood reflux is an important indicator of hemodynamic disturbance in patients with IVCS. Compression of the iliac vein can impede venous return, leading to localized retrograde flow and vortex formation (Xie et al., 2025). This disturbed flow field may promote local stasis within low-velocity recirculation zones, while elevated shear stress may arise in adjacent shear layers where forward and reverse flows interact and generate strong velocity gradients. These effects therefore occur in different spatial regions of the same abnormal hemodynamic environment, rather than representing a contradiction (Condemi et al., 2017). Moreover, gravitational effects can further influence venous return, particularly during prolonged inactivity, by contributing to hydrostatic pressure loading within the venous system. Such hemodynamic disturbances may be associated with symptoms such as leg heaviness and venous congestion. Therefore, it is important to investigate the role of collateral circulation in modulating blood reflux and other hemodynamic parameters in order to improve the mechanistic understanding of IVCS.

The porous medium model is a mathematical framework used to describe fluid flow through porous structures (Wang, 2000) and has been widely applied in Computational Fluid Dynamics (CFD) studies to simulate complex hemodynamic environments. In IVCS, this framework can be used to approximate the net flow-resistance effect of venous spur structures within the compressed venous segment. Venous spurs are intraluminal fibrotic septation-like formations that may develop as a consequence of chronic mechanical compression and repeated arterial pulsation. These structures partially obstruct the venous lumen, increase local hydraulic resistance, and disturb flow organization. In the present study, the compressed segment was represented using a porous-medium approach to capture the obstructive effect of venous spur-like structures while preserving computational tractability (Bear and Bachmat, 1990). This approach enables the simulation of increased resistance and flow asymmetry within the stenotic venous segment and provides a practical method for evaluating the hemodynamic consequences of compression.

Despite increasing clinical recognition of iliac vein compression syndrome (IVCS), the hemodynamic role of collateral circulation remains insufficiently understood, particularly from a quantitative fluid mechanics perspective. Previous CFD studies primarily focused on stenosis severity, wall shear stress, and pressure gradients in simplified or idealized venous geometries, whereas the functional impact of collateral pathways on flow redistribution, reflux formation, and energy dissipation has received limited attention. Accordingly, the present study was designed as an exploration, mechanistic, and hypothesis-generating CFD investigation. The specific scientific problem addressed here is how collateral pathways modify local disturbed-flow structures, pressure burden, and wall shear stress in patient-specific IVCS geometries. To address this gap, this study employs patient-specific CFD models with and without collateral circulation, incorporating a porous media representation of venous spur-like obstruction and gravity-inclusive physiological loading conditions. This framework allows a comparative analysis of flow structures, pressure distribution, and wall shear stress, with the aim of improving mechanistic understanding rather than establishing statistically generalizable clinical conclusions.

## 2 Methods

### 2.1 Ethics statement

This study was conducted in accordance with the Declaration of Helsinki (revised 2013). The Ethics Committee of Hefei University of Technology reviewed the study protocol and determined that it met the criteria for exemption from ethical review and approval, as only anonymized imaging data were used and no human or animal experiments were involved.

### 2.2 Data source

This study employed clinical medical imaging data from two patients diagnosed with IVCS at the North District of the First Affiliated Hospital of Anhui Medical University to examine the impact of collateral circulation on hemodynamic characteristics. Computed Tomography Angiography (CTA) images combined with clinical evaluations revealed that both patients exhibited pathological evidence of left iliac vein compression. However, a marked difference in the extent of collateral circulation development between the two cases provided a valuable basis for this comparative analysis.

In Patient A, CTA imaging revealed significant compression of the left iliac vein, which restricted blood flow from the left iliac vein to the inferior vena cava. However, due to long-term adaptive physiological responses, this patient had developed a well-established collateral circulation system, allowing blood to bypass the stenotic segment through alternative venous pathways, thereby maintaining a certain degree of venous return. The collateral vessels were clearly delineated in the imaging, indicating their maturity and functional significance in compensating for impaired blood flow.

In contrast, Patient B also exhibited compression of the left iliac vein; however, CTA imaging did not show any well-developed or distinct collateral circulation. This absence indicated that venous outflow in this patient primarily relied on the compressed main iliac vein pathway, with no effective alternative routes for blood diversion, leading to more severe venous stasis in the lower extremities.

### 2.3 Model and mesh generation

**FIGURE 1:**
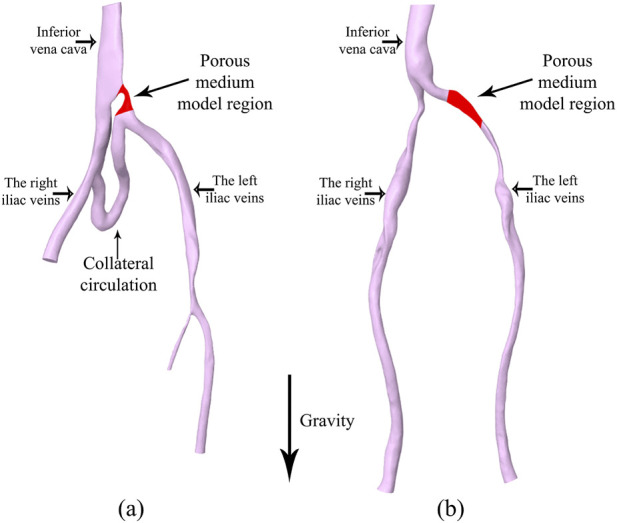
Reconstructed models of iliac vein compression: **(a)** collateral model: vascular system with collateral circulation pathways; **(b)** non-collateral model: vascular system without collateral circulation pathways.

In this study, high-fidelity three-dimensional vascular models were constructed from CTA data obtained from patients with IVCS. For Patient A, the reconstructed vascular system included the left and right iliac veins, collateral circulation pathways, and the inferior vena cava, forming the collateral model (Figure 1a). Blood enters the inferior vena cava from both the left and right iliac veins, with flow from the left iliac vein passing through both the stenotic segment and the collateral pathways, while flow from the right iliac vein proceeds directly into the inferior vena cava. In contrast, for Patient B, the lack of collateral pathways resulted in a vascular configuration consisting only of the left and right iliac veins and the inferior vena cava, forming the non-collateral model (Figure 1b). In this scenario, blood from the left iliac vein traverses the compressed segment before merging into the inferior vena cava, whereas blood from the right iliac vein flows directly into the inferior vena cava.

Unstructured tetrahedral meshes were generated for all models, with local refinement applied in the vicinity of the compressed region and adjacent collateral vessels. To ensure numerical reliability, a grid-independence study was performed using three progressively refined mesh resolutions of approximately 1 million, 2 million, and 4 million elements. These mesh levels were selected to span a practically relevant range for patient-specific iliac venous geometries while allowing assessment of convergence in the principal hemodynamic outputs. The pressure gradient between the left iliac vein and the inferior vena cava, together with the maximum wall shear stress in the stenotic segment, were selected as the primary evaluation metrics. The discrepancies between the medium- and fine-resolution meshes were less than 5%, indicating that further refinement produced only limited changes in the key hemodynamic quantities of interest and that the numerical solution was sufficiently mesh-independent for the purposes of the present study. Therefore, the medium-resolution mesh was adopted for the subsequent simulations to achieve an appropriate balance between computational efficiency and numerical accuracy. The final meshes used for the patient-specific collateral and non-collateral models contained approximately 2.35 million and 2.24 million elements, respectively. These final mesh sizes fall within the validated medium-resolution range established by the grid-independence study and were therefore considered sufficient for obtaining mesh-independent results.

### 2.4 Fluid model

This study investigated blood reflux, vortex formation and other disturbed-flow features using the Shear Stress Transport (SST) k-ω model to simulate blood flow dynamics. The SST k-ω model combines the advantages of both the k-ε and k-ω formulations and is well suited for flow fields involving separation, recirculation, and strong near-wall gradients (Sarfare et al., 2023).

To assess the local flow regime, the Reynolds number was estimated using the peak velocity and characteristic vessel diameter. In the stenotic segment, the Reynolds number approached transitional levels because of the locally elevated velocities (∼1.1 m/s), suggesting the possibility of flow instability. Although venous flow is generally laminar, stenotic geometries can generate flow separation, recirculation, and transitional flow features (Bluestein et al., 2000). Accordingly, the SST k-ω model was adopted to better represent these complex disturbed-flow characteristics, especially near-wall gradients and vortex structures (Anderson et al, 2000). No claim of fully developed turbulence is intended in this context. Thus, the use of the SST k-ω model in the present study is not meant to assume turbulent flow throughout the venous system, but rather to improve the numerical description of locally disturbed flow near the stenotic and post-stenotic regions.

The k-ε model is widely used in engineering applications for turbulent-flow simulation. However, its predictive accuracy may be limited in low-Reynolds-number and conditions (Bluestein et al., 2000). By contrast, the SST k-ω framework provides improved performance for near-wall flow and separated-flow regions, which are particularly relevant to the disturbed hemodynamics observed in compressed venous geometries. Furthermore, the SST model demonstrates good numerical stability and adaptability across different flow domains, which makes it appropriate for the present patient-specific simulations. (Zhang et al., 2013).

### 2.5 Porous medium model

A porous-medium model was adopted to represent the compressed segment of the iliac vein in both the collateral and non-collateral configurations. The porous medium region was defined on the basis of Digital Subtraction Angiography (DSA) imaging data and expert consultation with vascular clinicians (Figure 1), focusing on the portion of the left iliac vein exhibiting the most pronounced compression. The porosity of was set to be 1.0, and the viscous resistance coefficient was assigned a value of 2.5 × 10^8^, thereby representing a condition of severe iliac vein compression. The viscous resistance coefficient was adopted from Li et al. (2024), in which comparable porous medium parameters were calibrated against clinically observed hemodynamic characteristics of venous obstruction. Although a dedicated sensitivity analysis was not performed in the present study, previous work suggests that this parameter primarily influences the magnitude of flow redistribution rather than the overall qualitative flow pattern. Future studies should include a systematic sensitivity analysis of the porous-medium coefficients. Flow dynamics within the porous medium region were calculated using the Brinkman equation, which incorporates viscous and inertial resistance effects.

### 2.6 Gravity model

Venous hemodynamics may be influenced by multiple physiological factors, including hydrostatic loading associated with gravity. Previous studies have shown that body posture and gravity can affect venous pressure distribution and venous return under physiological conditions (Raju, 2019; Meissner et al., 2007; Modin and Shashkov, 2005; Sagirov et al., 2023). In the present study, gravity was included to preserve physiological realism by accounting for hydrostatic pressure distribution within the venous system under clinically relevant conditions. Gravity was treated as a background physiological loading condition rather than as an independent variable under investigation. Therefore, the current study does not attempt to isolate the specific contribution of gravity to the differences between the two anatomical models. A dedicated comparison of simulations with and without gravity would be required to quantify its independent hemodynamic effect and should be addressed in future work. Accordingly, the gravitational vector was oriented parallel to the inferior vena cava and opposite to the primary flow direction, with a magnitude of g = −9.8 m/s^2^ (Figure 1).

### 2.7 Boundary conditions

Both models employed similar boundary conditions to ensure comparability of the simulation results. The vessel walls were defined as no-slip walls. Blood was modeled as an incompressible Newtonian fluid with a dynamic viscosity of 0.0029 kg/(m·s) and a density of 1050 kg/m^3^. The left and right iliac veins were designated as velocity inlets, while the inferior vena cava was configured as a pressure outlet.

For the left iliac vein inlet, a time-dependent velocity function was prescribed based on clinically observed ultrasound spectrogram patterns and simplified as follows: $v=0.108+0.0835\;*\;\sin\;\left(1.5t-1.5\right)\;m/s$ . The time-dependent inlet waveform was derived from clinically reported Doppler ultrasound patterns of iliac venous flow and was used as a simplified representation of the respiratory modulation of venous return. The characteristic period of approximately 4.2 s therefore does not represent a cardiac cycle, but rather a respiratory-related inflow variation, which is physiologically relevant for lower-extremity venous return. The imposed velocity pattern had a period of approximately 4.2 s, corresponding primarily to respiratory-related variation in venous return rather than cardiac pulsation.

For the right iliac vein inlet, a constant inlet velocity of 0.099 m/s was prescribed to represent the mean venous flow condition. The left iliac vein was assigned a time-dependent waveform because it was the vessel directly affected by compression and therefore constituted the primary focus of the present hemodynamic analysis. By contrast, the right iliac vein inlet was simplified as a constant mean inflow in order to represent its average contribution to systemic venous return while minimizing additional uncertain boundary-condition inputs. This modeling choice was intended to focus the analysis on the disturbed hemodynamics associated with the compressed left iliac vein rather than to claim a fully patient-specific bilateral inflow condition. We acknowledge that this assumption may influence global flow distribution and does not fully reproduce physiological temporal variation; therefore, it is treated as a model limitation.

Sensitivity analysis indicated that perturbations in the right inflow produced negligible changes in velocity magnitude, pressure gradient, and wall shear stress within the compressed region. Therefore, this simplification was considered acceptable for the mechanistic comparisons performed in the present study, although it should not be interpreted as a fully physiological representation of bilateral inflow.

The inferior vena cava outlet was assigned a pressure boundary condition with a static pressure of 5 mmHg (approximately 650 Pa), consistent with clinically physiologically realistic central venous pressure levels.

Given that the left iliac vein inlet velocity function exhibits a periodic cycle of 4.2 s, the transient simulation was conducted over two full cycles (8.4 s total) to capture the principal flow dynamics. A time step of 0.02 s was employed.

For all transient simulations, convergence criteria were applied at each time step, with the residuals of the continuity, momentum, and turbulence model equations required to fall below 10^-3^ to ensure numerical stability and solution reliability. To assess temporal resolution, a time-step independence study was performed using additional time steps of 0.01 s and 0.04 s. Key hemodynamic metrics—including the pressure gradient between the left iliac vein and the inferior vena cava, the maximum velocity within the compressed region, and the peak wall shear stress—were compared across all three time step settings. The discrepancies between the results obtained with 0.02 s and 0.01 s were less than 1%, indicating that a time step of 0.02 s provided adequate temporal accuracy while preserving computational efficiency.

## 3 Results

### 3.1 Reflux analysis

**FIGURE 2:**
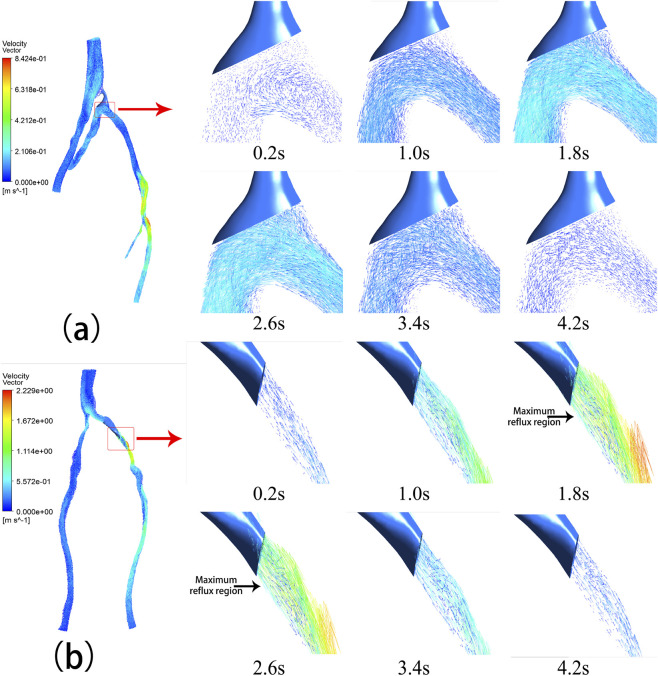
Distribution of reverse blood flow over time: **(a)** Collateral model; **(b)** Non-collateral model. Reverse flow was identified using streamline orientation, with arrow direction indicating local flow direction. The figure presentation and caption were revised to improve the visibility of reflux patterns and recirculating flow structures.

Under the combined influence of the porous-medium obstruction and the applied physiological loading conditions, blood reflux was observed in both the collateral and non-collateral models. In the collateral model (Figure 2a), blood primarily bypassed the obstructed region via collateral pathways to reach the inferior vena cava, with only localized reflux occurring at the distal end of the compressed segment. This localized reflux quickly integrated into the collateral vessels, forming smaller vortices that had minimal impact on the overall flow pattern. In contrast, in the non-collateral model (Figure 2b), blood was forced to pass through the compressed left iliac vein to reach the inferior vena cava, resulting in the formation of larger reflux and recirculating structures within the left iliac vein.

**FIGURE 3:**
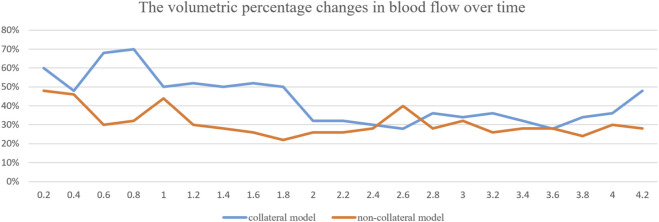
Comparison of the volumetric percentage changes in blood flow from the left iliac vein to the inferior vena cava between collateral model and non-collateral model.

The line graph shown in Figure 3 quantitatively illustrates this difference, highlighting a substantial discrepancy in blood flux from the left iliac vein to the inferior vena cava between the two models. Figure 3 demonstrates that the volumetric percentage of blood flowing into the inferior vena cava via collateral pathways in the collateral model was significantly higher than the passing through the compressed left iliac vein in the non-collateral model. Over the course of two complete cycles, the total volume of blood returning to the inferior vena cava was approximately 39.38% greater in the collateral model, with the average blood flow per time step being approximately 43.4% higher.

**FIGURE 4:**
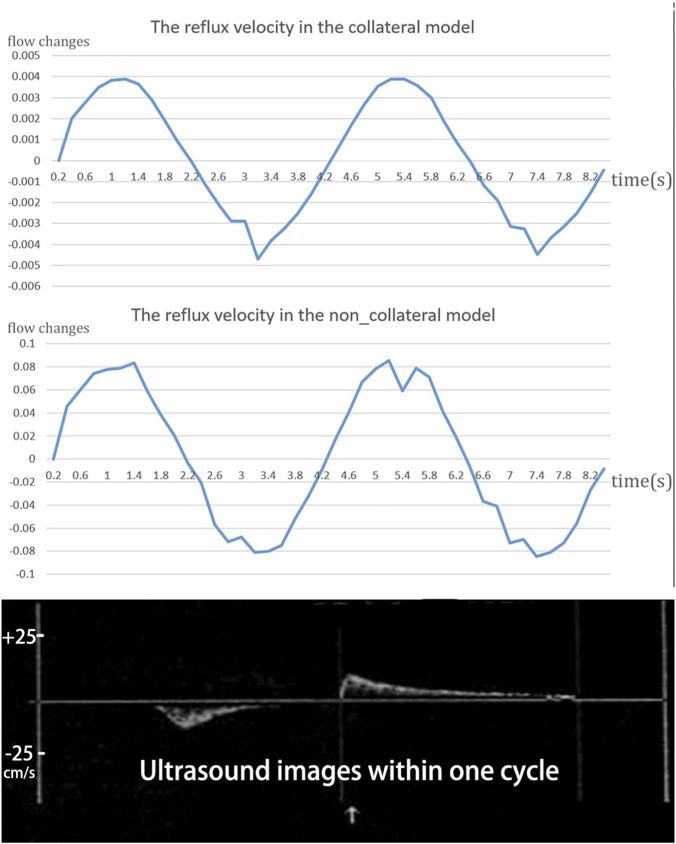
Comparison of the reflux waveform extracted from the CFD simulation with the Doppler ultrasound spectral pattern obtained from the corresponding patient-specific anatomical region.

Additionally, a temporal waveform plot of the reflux was generated at the distal location relative to the stenosis, where reflux was most prominent. This waveform was subsequently compared with clinical ultrasound waveforms obtained from the corresponding anatomical region. As shown in Figure 4, reflux in both the model with collateral circulation and the model without collateral circulation exhibited strong temporal dependence and closely resembled the reflux patterns observed clinically. Notably, the magnitude of reflux in the non-collateral model was significantly greater than that in the collateral model, reaching a peak value approximately 20 times higher at the point of maximum reflux.

Vortex formation of is influenced by vascular geometry and variations in blood flow velocity. In the present models, the disturbed-flow structures were primarily associated with the obstructive effect of the compressed porous segment, which generated localized resistance, recirculation, and flow separation. In the collateral model, diversion of blood through alternative venous pathways reduced the amount of flow forced through the compressed segment, thereby limiting reflux and vortex formation. In contrast, in the non-collateral model, the absence of alternative outflow pathways forced blood through the constricted region, leading to pronounced reflux and larger recirculating structures. These abnormal flow features may contribute to impaired venous return and to localized alterations in pressure and wall shear stress, but they should be interpreted as mechanistic hemodynamic observations rather than direct evidence of thrombus formation.

### 3.2 Flow velocity analysis

**FIGURE 5:**
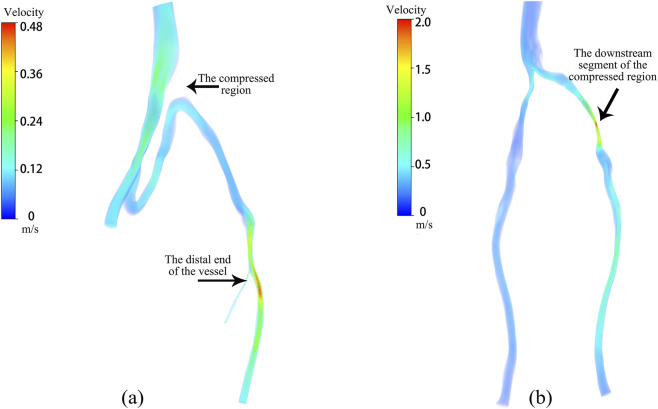
Distribution of blood flow velocity at 2.1 s: **(a)** Collateral model; **(b)** Non-collateral model.

As illustrated in Figure 5a, the collateral model exhibited a relatively uniform velocity distribution, with only a few localized regions of elevated velocity observed at the distal end of the vessel. The minimal blood flow through the compressed region, combined with the majority of blood being redirected through collateral vessels to the inferior vena cava, resulted in the absence of significant abnormal flow patterns.

In contrast, the non-collateral model demonstrated a clear temporal dependence of blood flow velocity within the compressed region (Figure 5b). At 2.1 s, the velocity of blood entering the compressed segment was approximately 1.11 m/s, which subsequently decreased to 0.36 m/s by 4.2 s. Conversely, in the collateral model, the velocity of blood entering the collateral vessels was 0.2 m/s at 2.1 s, and further declined to 0.04 m/s at 4.2 s.

Within the non-collateral model, higher-velocity blood flow was predominantly concentrated in the downstream segment of the compressed region (Figure 5b). The requirement for blood to pass through the porous medium in order to reach the inferior vena cava resulted in a relatively stable overall flow velocity despite the narrowed lumen. However, the marked increase in local velocity may contribute to stronger flow separation, localized vortex formation, and more pronounced disturbed-flow features within the compressed region.

These findings support the previous analysis indicating that collateral circulation not only improves flow redistribution but also mitigates abnormal hemodynamic behavior within the compressed segment. In the non-collateral model, exclusive reliance on the compressed channel for venous return, together with increased local velocity and localized vortex formation, may create a more adverse hemodynamic environment characterized by stasis-prone recirculation and abnormal wall shear stress gradients. However, this should not be interpreted as direct evidence of thrombus formation, which was not modeled in the present study.

### 3.3 Iliac vein pressure analysis

**FIGURE 6:**
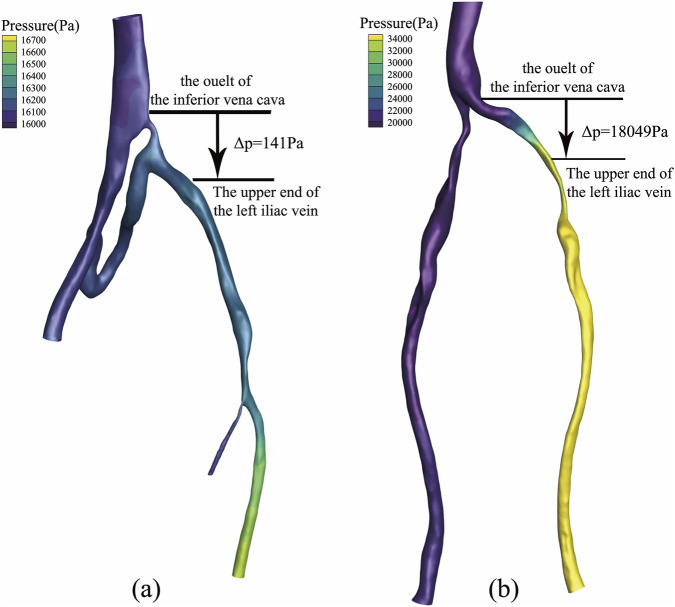
Pressure gradient distribution map at 2.1 s: **(a)** collateral model; **(b)** non-collateral model.

In the collateral model, an increase in inlet velocity during the interval of 0–2.1 s interval led to a corresponding rise in pressure within the left iliac vein, which reached its peak at 2.1 s (see Figure 6a). At this peak, the pressure gradient between the maximum pressure observed in the left iliac vein and the outlet of the inferior vena cava was measured at 619 Pa, while the pressure difference between the upper end of the left iliac vein and its outlet was recorded at 141 Pa (Figure 6a). Throughout the entire cycle, the pressure difference fluctuated within a range of 0–141 Pa, a relatively low range, indicating that collateral circulation effectively redirected a portion of the blood flow and thereby reduced flow resistance in the compressed region.

In contrast, in the model devoid of collateral circulation, the pressure in the left iliac vein also increased with the inlet velocity, peaking at 2.1 s (Figure 6b). However, at this point, the pressure gradient between the maximum pressure in the left iliac vein and the outlet of the inferior vena cava was as high as 20,190 Pa (approximately 151 mmHg), with the pressure difference between the upper end of the left iliac vein and the outlet recorded at 18,049 Pa. Over the course of the entire cycle, the pressure difference varied from 2,312 to 18,049 Pa, significantly exceeding the pressure levels observed in the collateral model.

The elevated pressure difference observed in Figure 6b is partly attributable to the increased length of the compressed segment, as a longer stenosis enhances viscous energy dissipation along the flow direction. However, under the geometric dimensions and hemodynamic conditions considered in this study, elongation of the stenotic segment alone cannot fully account for the magnitude of the observed pressure difference.

Instead, the pronounced pressure elevation is primarily driven by the substantial reduction in effective flow area—and the consequent sharp increase in hydraulic resistance—within the compressed region. This effect is further amplified by the porous medium representation of the stenosis. As a result, blood flow is marked redistributed toward collateral pathways, leading to a nonlinear increase in the pressure gradient between the left iliac vein and the inferior vena cava.

These findings demonstrate that collateral circulation can effectively reduce flow resistance in the compressed region, thereby maintaining lower pressure levels within the iliac vein. In contrast, the absence of collateral circulation forces blood through the compressed region, resulting in substantial flow restrictions and a marked elevation in local venous pressure. Such pressure elevation, when considered together with flow stasis, prolonged residence time in recirculation zones, and abnormal wall shear stress gradients, may contribute to a disturbed hemodynamic environment that is mechanistically associated with pro-thrombotic conditions. According to Virchow’s triad, these features may promote endothelial dysfunction and local activation of coagulation pathways. Nevertheless, direct thrombosis was not simulated in the present study, and these observations should therefore be interpreted as mechanistic rather than predictive.

### 3.4 Wall shear stress (WSS) analysis

**FIGURE 7:**
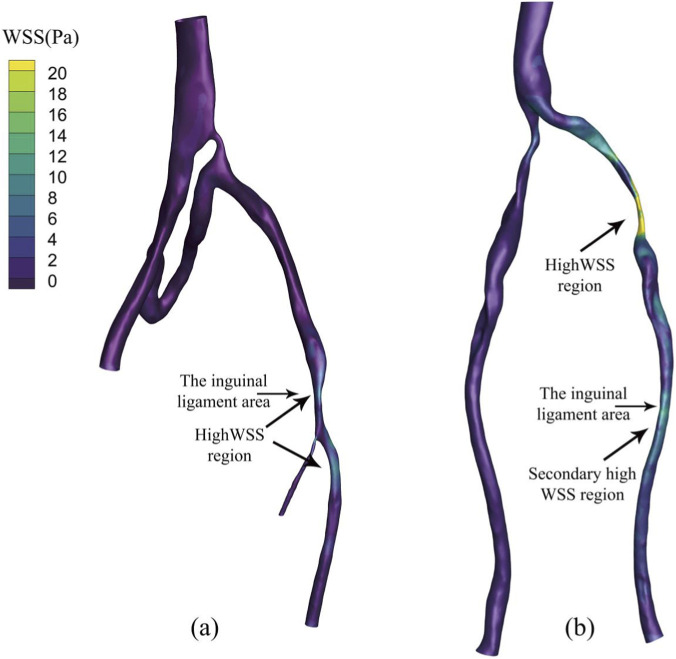
WSS distribution at 2.1 s: **(a)** collateral model; **(b)** non-collateral model.

In the collateral model (Figure 7a), regions of elevated WSS were predominantly localized in the constricted lower segment of the vein, with a peak WSS of approximately 10 Pa. The WSS observed downstream of the compressed iliac vein region was approximately 3 Pa, indicating that the vessel walls in this area experienced relatively low shear forces.

In contrast, in the non-collateral model (Figure 7b), multiple regions of elevated WSS were identified within the left iliac vein, with a maximum WSS of 56 Pa—approximately 5.6 times higher than that observed in the collateral model. The average WSS downstream of the compressed region in the non-collateral model was approximately 30 Pa, representing a tenfold increase compared to the collateral model. These findings indicate that venous stenosis leads to an increase in local shear stress, regardless of the presence of collateral circulation. However, in the absence of collateral pathways, both the peak WSS in the stenotic region and the average WSS downstream of the compressed segment were significantly higher than those in the collateral model. Rather than interpreting these findings as direct evidence of thrombosis or wall injury, we consider them to reflect an adverse hemodynamic environment characterized by abnormal mechanical loading on the vessel wall (Zhou et al., 2023). Such loading may be mechanistically relevant to endothelial dysfunction and vascular remodeling (Metaxa et al., 2010; Stefanadis et al., 2017), although these biological processes were not directly modeled in the present study.

## 4 Discussion

This study utilized computational fluid dynamics (CFD) to perform a comparative analysis of the hemodynamic characteristics in two patient-specific models with Iliac Vein Compression Syndrome (IVCS), one with collateral circulation and one without. The present findings should be interpreted as exploratory and mechanistic rather than predictive. Within this scope, the results suggest that collateral circulation may function as an important compensatory pathway that redistributes venous flow and modifies local reflux, vortex formation, venous pressure, and wall shear stress (WSS). Because only two patient-derived anatomical models were analyzed, the observed quantitative differences should be understood as case-specific hemodynamic observations rather than generalizable clinical outputs.

### 4.1 The role of collateral circulation in modulating hemodynamic disturbances in IVCS

This study identifies two distinct hemodynamic patterns associated with IVCS, with the principal difference being the presence or absence of effective collateral circulation. In the non-collateral model, the left common iliac vein serves as the sole major pathway for venous return. Following compression and stenosis, outflow resistance increases substantially, resulting in elevated local pressure, more prominent reflux, and larger recirculating flow structures distal to the stenosis site. These disturbed-flow features may reduce the efficiency of venous return and promote localized blood stasis-prone regions, thereby creating a hemodynamic environment that may be mechanistically associated with thrombus-prone conditions, although thrombus formation itself was not modeled (Wolberg et al., 2015).

In contrast, in the collateral model, the collateral vessel network provides an effective alternative pathway, diverting a substantial portion of blood flow away from the compressed left common iliac vein. This redistribution reduces the flow burden through the stenotic segment and consequently alleviates local pressure buildup and disturbed-flow intensity. As a result, reflux and vortex formation are cinfined to a smaller region, yielding a comparatively more stable hemodynamic environment. These findings support the interpretation that collateral circulation functions as an adaptive compensatory mechanism in response to venous outflow obstruction. From a hemodynamic perspective, this pressure-driven diversion of flow toward collateral channels may be viewed as a steal-like redistribution phenomenon, in the sense that collateral pathways offload the high-resistance stenotic route. However, the present study does not attempt to define or quantify a formal vascular steal syndrome, and the observation should therefore be interpreted cautiously as flow redistribution captured by the patient-specific CFD models. Because the present analysis is based on only two patient-specific models, the conclusions should be interpreted as exploratory and hypothesis-generating rather than broadly generalizable.

### 4.2 Association between hemodynamic changes and vascular wall pathological injury

The venous pressure gradient is an important hemodynamic parameter in IVCS. In the present study, the pressure gradient in the stenotic region of the non-collateral model was markedly higher than that in the collateral model, indicating substantially greater resistance to venous outflow. This local venous hypertension may contribute to chronic venous dysfunction and may help explain some of the clinical manifestations associated with impaired venous drainage (Travers et al., 1996).

Abnormal wall shear stress is another important biomechanical biomechanical factor relevant to vascular pathology. In the non-collateral model, elevated WSS was observed both at the site of stenosis and in the downstream disturbed-flow region. Rather than interpreting these findings as direct evidence of thrombosis or wall injury, we consider them to reflect an adverse hemodynamic environment characterized by abnormal mechanical loading on the vessel wall. Such loading may be mechanistically relevant to endothelial dysfunction and vascular remodeling, although these biological processes were not directly modeled in the present study. The findings therefore suggest that elevated pressure, disturbed flow, and abnormal WSS may collectively contribute to unfavorable local hemodynamic conditions in IVCS.

### 4.3 Implications for clinical diagnosis and treatment

Because no treatment scenarios were directly simulated in the present study, the implications of these findings should be limited primarily to mechanistic interpretation rather than therapeutic recommendation. Within this scope, the results suggests that the assessment of collateral circulation may provide useful complementary information beyond stenosis severity alone, because collateral pathways can substantially alter flow redistribution, reflux extent, local pressure burden, and wall shear stress patterns. Accordingly, these CFD observations may help improve physiological understanding of why patients with apparently similar degrees of anatomical compression can exhibit different hemodynamic environments. Further work incorporating larger patient cohorts, treatment simulations, and outcome data will be required before any diagnostic or therapeutic conclusions can be drawn.

### 4.4 Limitations and future perspectives

This study has several limitations. First, the model assumed rigid vessel walls and therefore neglected vascular deformation under physiological pressure loading. Future work could incorporate fluid-structure interaction (FSI) to better represent vessel-wall mechanics. Second, the anatomical structures used in this study were derived from only two patient-specific cases, whereas substantial inter-individual variability exists in both venous anatomy and collateral pathway development. This limitation was not fully overcome in the present work because patient-specific reconstruction of complex venous and collateral anatomies is labor-intensive, and the acquisition of sufficiently detailed imaging and flow data for a larger cohort remains challenging. Therefore, the present findings should be interpreted as exploratory and hypothesis-generating rather than statistically generalizable. Third, blood was modeled as a Newtonian fluid, which is a common simplification in large-vessel hemodynamics. However, non-Newtonian effects may become more important in low-shear regions such as reflux and recirculation zones. Finally, fully quantitative model validation was limited by the absence of synchronized high-resolution patient-specific flow measurements. The comparison between simulated reflux waveforms and clinical ultrasound was therefore qualitative and intended to assess consistency in temporal pattern rather than provide full quantitative validation. Future studies should incorporate larger and more diverse patient cohorts, more comprehensive physiological boundary conditions, and additional biological or clinical validation.

Overall, the present study should be interpreted as a case-based mechanistic analysis intended to illustrate plausible hemodynamic tendencies in IVCS, rather than to predict clinical outcomes or establish population-level quantitative benchmarks.

## 5 Conclusion

Through a comparative analysis of IVCS models with and without collateral circulation, this study suggests that collateral pathways may play an important hemodynamic compensatory role by redistributing venous flow and reducing pressure burden, wall shear stress, and the extent of disturbed-flow structures within the compressed region. In contrast, the absence of collateral compensation was associated with more severe reflux, localized vortex formation, elevated pressure gradients, and abnormal wall shear stress distributions. While the specific spatial distribution and magnitude of reflux and vortex structures are inherently dependent on individual vascular geometry, the hemodynamic mechanism identified here should be interpreted as a mechanistic observation derived from these patient-specific models rather than as a universal hemodynamic law for all IVCS cases. These findings should be interpreted as mechanistic and hypothesis-generating rather than as direct evidence of thrombotic risk or clinical treatment effect. The present study therefore supports the view that collateral circulation is an important determinant of the local hemodynamic environment in IVCS, while also highlighting the need for validation in larger patient cohorts and with more comprehensive physiological and biological modeling.

## Data Availability

The raw data supporting the conclusions of this article will be made available by the authors, without undue reservation.
